# Identifying Potential Antioxidant Properties from the Viscera of Sea Snails (*Turbo cornutus*)

**DOI:** 10.3390/md19100567

**Published:** 2021-10-13

**Authors:** Nalae Kang, Eun-A Kim, Junseong Kim, Seung-Hong Lee, Soo-Jin Heo

**Affiliations:** 1 Jeju Marine Research Center, Korea Institute of Ocean Science and Technology (KIOST), Jeju 63349, Korea; nalae1207@kiost.ac.kr (N.K.); euna0718@kiost.ac.kr (E.-A.K.); junseong@kiost.ac.kr (J.K.); 2 Department of Pharmaceutical Engineering and Medical Science, Soonchunhyang University, Asan 31538, Korea; 3 Department of Biology, University of Science and Technology (UST), Daejeon 34113, Korea

**Keywords:** *Turbo cornutus*, viscera, antioxidant, hydrogen peroxide, bioactive peptide

## Abstract

*Turbo cornutus*, the horned turban sea snail, is found along the intertidal and basaltic shorelines of Jeju Island, Korea. *T. cornutus* feeds on seaweeds (e.g., *Undaria* sp., and *Ecklonia* sp.) composed of diverse antioxidants. This study identified potential antioxidant properties from *T. cornutus* viscera tissues. Diverse extracts were evaluated for their hydrogen peroxide (H_2_O_2_) scavenging activities. *T. cornutus* viscera protamex-assisted extracts (TVP) were purified by gel filtration chromatography (GFC), and potential antioxidant properties were analyzed for their amino acid sequences and its peroxidase inhibition effects by in silico molecular docking and in vitro analysis. According to the results, *T. cornutus* viscera tissues are composed of many protein contents with each over 50%. Among the extracts, TVP possessed the highest H_2_O_2_ scavenging activity. In addition, TVP-GFC-3 significantly decreased intracellular reactive oxygen species (ROS) levels and increased cell viability in H_2_O_2_-treated HepG2 cells without cytotoxicity. TVP-GFC-3 comprises nine low molecular bioactive peptides (ELR, VGPQ, TDY, ALPHA, PAH, VDY, WSDK, VFSP, and FAPQY). Notably, the peptides dock to the active site of the myeloperoxidase (MPO), especially TDY and FAPQY showed the MPO inhibition effects with IC_50_ values of 646.0 ± 45.0 µM and 57.1 ± 17.7 µM, respectively. Altogether, our findings demonstrated that *T. cornutus* viscera have potential antioxidant properties that can be used as high value-added ingredients.

## 1. Introduction

*Turbo cornutus*, an edible gastropod species with a horned turban, is found along the intertidal and basaltic shorelines of Jeju Island, Korea. *T. cornutus* is a major source of income for Jeju Haenyeo (women divers; Intangible Cultural Heritage, 2016). The muscle tissues of *T. cornutus* are used as local foods in Jeju, Korea, but most of its viscera tissues are discarded because of low consumer preference and awareness. Some studies published in the 1970s–2000s presented the physioecology of *T. cornutus* [1,2,3]. *T. cornutus* is an herbivorous marine animal that feeds on seaweeds composed of diverse antioxidants [4]. However, the nutritional and functional ingredients of *T. cornutus* remain unknown.

Under normal physiological conditions, intracellular reactive oxygen species (ROS) are maintained at a constant low level by the balance between the generation and elimination of ROS [5]. However, ROS generated without control can induce oxidative damage to intracellular biomacromolecules, such as protein, membrane lipid, RNA, and DNA [5,6]. Hydrogen peroxide (H_2_O_2_) is a ROS that, when present in excess, can be harmful to cells [7]. In addition, H_2_O_2_ can be converted the hypohalous acids, causing oxidative damage by the Myeloperoxidase (MPO)/H_2_O_2_ system [8,9]. Thus, the removing H_2_O_2_ is very important to combat oxidative stress and MPO-dependent ROS [10,11]. 

Several antioxidants prevent and relieve oxidative damage caused by ROS [12]. Exogenous antioxidants are widely distributed in food and medicinal plants and food processing by-products, such as seafood viscera [13,14,15,16]. From this, many studies are being conducted on search for natural antioxidant compounds.

Yearly, a considerable amount of world fishery resources are discarded as processing leftovers, such as viscera, gonads, bones, and skin [17]. These marine by-products cause problems, such as environmental pollution. Thus, efforts to explore the possibilities for the further use of marine by-products have become more important than the methods of their disposal [18,19,20,21]. Recently, much research is conducted to explore the possible uses of different by-products; many studies have presented that marine by-products contain valuable protein fractions, including surimi [22], gelatin/collagen [23], and bioactive peptides [24]. Producing functional food materials and other value-added products from marine by-products is a way to obtain attention because marine by-products contain valuable protein and lipid fractions, pigments, minerals, enzymes, and nutraceuticals or pharmacological [18,20]. 

The objective of this study is to explore valuable application process that can reuse the discarded viscera of *T. conutus*. The potential antioxidant properties were purified from *T. cornutus* viscera through enzymatic hydrolysis and gel filtration chromatography (GFC); also, its antioxidant activities were assessed in H_2_O_2_-treated HepG2 cells. Furthermore, the bioactive peptides that composed the potential antioxidant properties were analyzed for their peroxidase inhibition effect.

## 2. Results and Discussion

### 2.1. Proximate Composition of T. cornutus

The proximate composition of each *T. cornutus* viscera and muscle is shown in Table 1. Protein was the major chemical component of the *T. cornutus* viscera; protein contents accounted for 52.68% ± 0.28% of the total dry weight. The lipid, moisture, ash, and carbohydrate contents of *T. cornutus* viscera were 28.40% ± 1.20%, 1.03% ± 0.35%, 14.79% ± 0.80%, and 3.12% ± 1.93%, respectively. The major chemical component of the *T. cornutus* muscle was protein; protein contents accounted for 78.28% ± 2.23% of the total dry weight. The lipid, moisture, ash, and carbohydrate contents of *T. cornutus* muscle were 10.90% ± 0.81%, 4.25% ± 3.66%, 4.86% ± 0.40%, and 1.73% ± 1.85%, respectively. Thus, *T. cornutus* viscera and muscle are rich protein sources.

### 2.2. Amino Acid Composition of T. cornutus

The amino acid compositions of *T. cornutus* viscera and muscle are listed in Table 2. The most abundant amino acids in the *T. cornutus* viscera are aspartic acid (10.3 ± 0.0%), glutamic acid (13.1 ± 0.2%), and taurine (11.3 ± 0.1%), each of which comprises more than 10% of the *T. cornutus* viscera, followed by arginine (7.1±0.5%), leucine (6.5 ± 0.1%), and proline (6.2 ± 0.3%). Alternatively, the most abundant amino acids in the *T. cornutus* muscle are aspartic acid (9.5 ± 0.2%), glutamic acid (16.4±0.1%), and arginine (9.6 ± 0.0%), followed by glycine (8.8 ± 0.2%), taurine (8.1 ± 0.0%), and leucine (6.3 ± 0.1%). Aspartic acid, glutamic acid, arginine, and glycine are the most abundant amino acids in other marine animals, such as abalone [25]. Both the viscera and muscle contain the most abundant aspartic and glutamic acid. The viscera and muscle contain about 30% of the essential amino acid composition, such as histidine, threonine, valine methionine, phenylalanine, isoleucine, leucine, and lysine, for humans. Therefore, both of them are high-quality protein sources. 

### 2.3. H_2_O_2_ Scavenging Activity of the Enzymatic Extracts of T. cornutus

**Figure 1:**
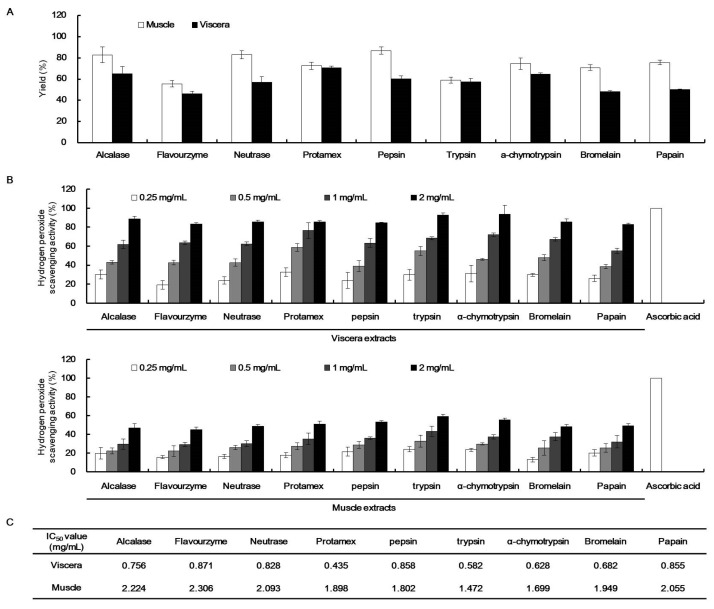
Extraction yields and hydrogen peroxide (H_2_O_2_) scavenging activities of *Turbo cornutus* extracts. The extraction yields (**A**), H_2_O_2_ scavenging activities (**B**), and IC_50_ values (**C**) of each *T. cornutus* viscera and muscle enzyme-assisted extract. (**C**) IC_50_ values on H_2_O_2_ scavenging activities of *Turbo cornutus* extracts.

To assess the H_2_O_2_ scavenging activity of *T. cornutus* viscera and muscle, each viscera and muscle tissues was hydrolyzed with nine proteases: alcalase, flavourzyme, neutrase, protamex, pepsin, trypsin, α-chymotrypsin, bromelain, and papain. The extraction yields of diverse *T. cornutus* enzyme-assisted extracts are summarized in Figure 1A. The *T. cornutus* viscera enzyme-assisted extracts showed more than 40% extraction yields, with *T. cornutus* viscera protamex-assisted extracts (TVP) having the highest extraction yield (70%). In contrast, the *T. cornutus* muscle enzyme-assisted extracts showed higher extraction yields than those of the viscera.

The H_2_O_2_ scavenging activities of the enzymatic extracts of *T. cornutus* viscera and muscle were shown in Figure 1B,C. The viscera extracts indicated higher H_2_O_2_ scavenging activities than those of the muscle extracts in a concentration ranging from 0.25 to 2 mg/mL. The viscera extracts showed more than 80% H_2_O_2_ scavenging activities at 2 mg/mL. In addition, the viscera extracts showed approximately three times higher IC_50_ values of H_2_O_2_ scavenging activities against each muscle extract, with TVP having the lowest IC_50_ value of 0.435 mg/mL.

### 2.4. Effect of Viscera and Muscle Extracts on H_2_O_2_-Induced Oxidative Stress in HepG2 Cells

**Figure 2:**
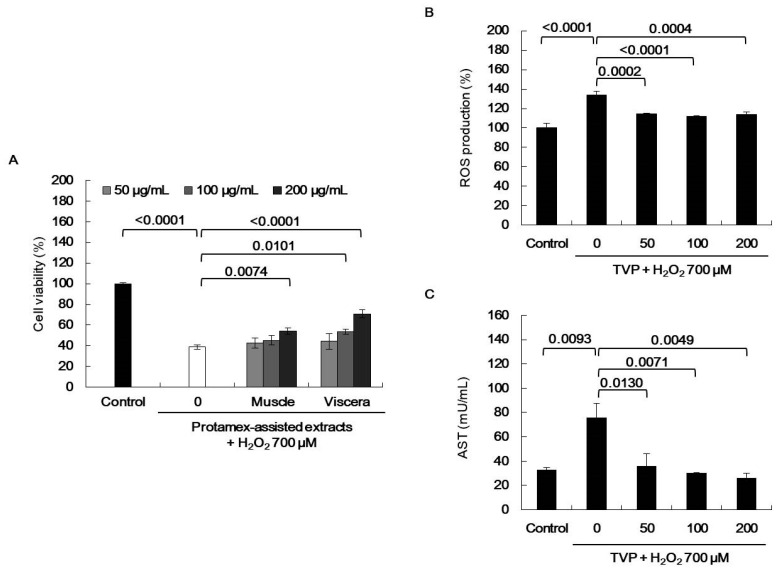
Effects of *Turbo cornutus* extracts on hydrogen peroxide (H_2_O_2_)-induced oxidative stress in HepG2 cells. (**A**) Protective effect of each *T. cornutus* muscle and viscera protamex extracts on oxidative stress in HepG2 cells. (**B**) Intracellular reactive oxygen species production inhibition effects of *T. cornutus* viscera protamex extracts (TVP) on oxidative stress in HepG2 cells. (**C**) Aspartate aminotransferase (AST) production inhibition effects of TVP on oxidative stress in HepG2 cells. The data are expressed as means ± standard deviation (SD) of three determinations.

The liver is a vital organ that plays a major role in metabolism, excretion, and detoxification in the human body. Liver impairment is caused by different factors, such as infection, drugs, and excessive ethanol intake, leading to the accumulation of ROS and various liver injuries by oxidative stress. Thus, oxidative stress prevention is needed for hepatoprotection [26]. ROS are broadly defined as chemically reactive molecules containing oxygen; these include hydroxyl radical (OH·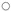), superoxide anion (O_2_^−^), singlet oxygen (O_2_), and H_2_O_2_ (H_2_O_2_) [5]. ROS react with molecules by reversible oxidative modifications and factors in cellular signaling pathways, such as metabolism, growth, differentiation, and death signaling [5]. However, ROS overproduction without control can result in oxidative damage to cell structures, including lipids and membranes, proteins, and DNA [5,26]. Therefore, the MTT assay was performed in H_2_O_2_-exposed HepG2 cells to evaluate the potential antioxidant effect of viscera and muscle extract s. As shown in Figure 2, significant cell death was observed in the H_2_O_2_-treated cells. However, TVP and the muscle protamex extract markedly increased cell viability. Especially, TVP showed a higher protective effect than did muscle protamex-assisted extracts against H_2_O_2_ in HepG2 cells. In addition, TVP inhibited intracellular ROS production, and aspartate aminotransferase (AST) levels increased by treating H_2_O_2_ in HepG2 cells. These results indicated that *T. cornutus* viscera tissues possess a high value than did *T. cornutus* muscle tissues by protamex-assisted hydrolysis processing.

### 2.5. Characterization of Antioxidant Peptides from TVP

**Figure 3:**
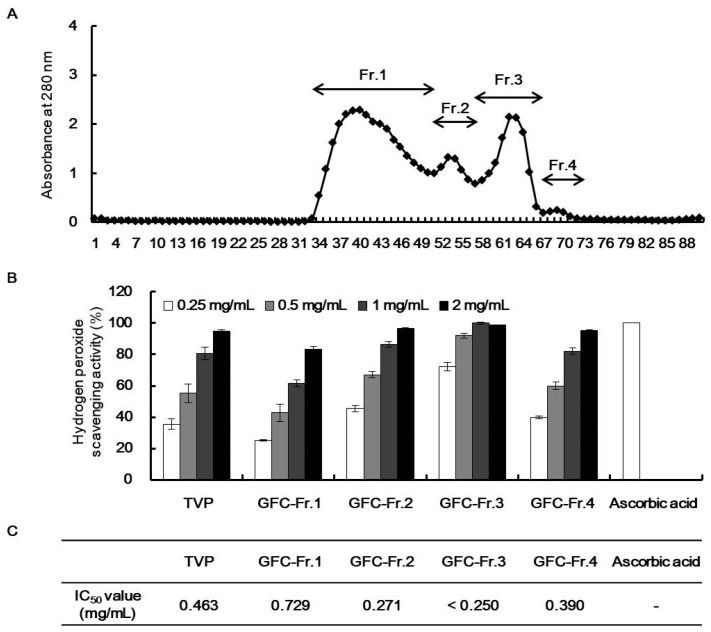
Hydrogen peroxide (H_2_O_2_) scavenging activities of *Turbo cornutus* viscera protamex extract gel filtration chromatography fractions. (**A**) Gel filtration chromatogram of *T*. *cornutus* viscera protamex extracts using Sephadex G-25. H_2_O_2_ scavenging activities (**B**) and IC_50_ values (**C**) of each fraction.

**Figure 4:**
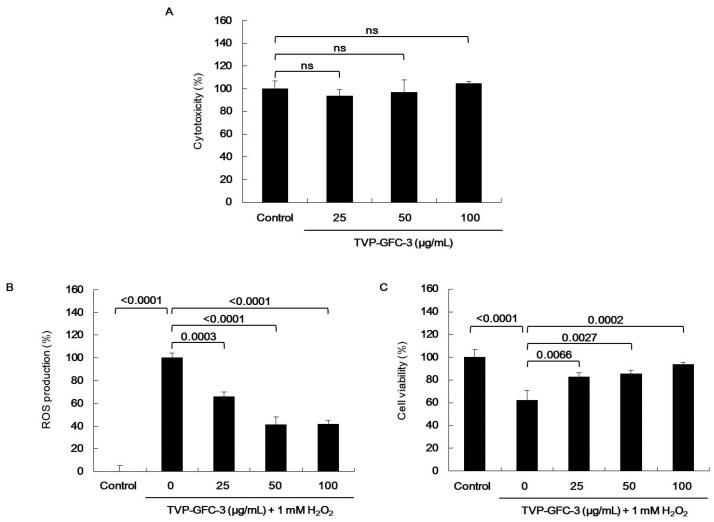
Effects of *Turbo cornutus* viscera protamex extract gel filtration chromatography (GFC) fractions on H_2_O_2_-induced oxidative stress in HepG2 cells. (**A**) Cytotoxicity of *T. cornutus* viscera protamex extract GFC fraction 3. (**B**) Intracellular reactive oxygen species production inhibition effects of *T. cornutus* viscera protamex extract GFC fraction 3 on oxidative stress in HepG2 cells. (**C**) AST production inhibition effects of *T. cornutus* viscera protamex extract GFC fraction 3 on oxidative stress in HepG2 cells. The data are expressed as means ± standard deviation (SD).

Depending on the H_2_O_2_ scavenging activity and protective effect on H_2_O_2_ in HepG2 cells, TVP was selected for the next separation step by GFC on Sephadex G-25 column. TVP was fractionated to four kinds of fractions according to their molecular sizes (Figure 3A). Among them, GFC-Fr.3 (TVP-GFC-3) had the highest H_2_O_2_ scavenging activity at a concentration of 0.25 mg/mL (Figure 3B,C). TVP-GFC-3 significantly increased IC_50_ values than did TVP. In addition, TVP-GFC-3 significantly decreased ROS generation and increased protective effects in H_2_O_2_-exposed HepG2 cells without cytotoxicity (Figure 4). To identify the amino acid sequences of the separated fraction, TVP-GFC-3 was analyzed using MicroQ–time-of-flight (TOF) tandem mass spectrometry. TVP-GFC-3 comprises nine small molecule peptides, and the amino acid sequences of the peptides were evaluated as ELR, VGPQ, TDY, ALPHA, PAH, VDY, WSDK, VFSP, and FAPQY (Table 3, Figures S1–S10).

### 2.6. In Silico Analysis of Antioxidant Peptides on MPO Inhibition

Several molecular docking studies targeting specific enzyme inhibition effects have been recently published [27,28]. Among the docking tools, CDOCKER, a CHARMm-based docking algorithm [29], found favorable docking poses between small molecules and target proteins using their structural characteristics, such as unshared electron pairs, double bonds, hydrophobicity, and charge.

**Figure 5:**
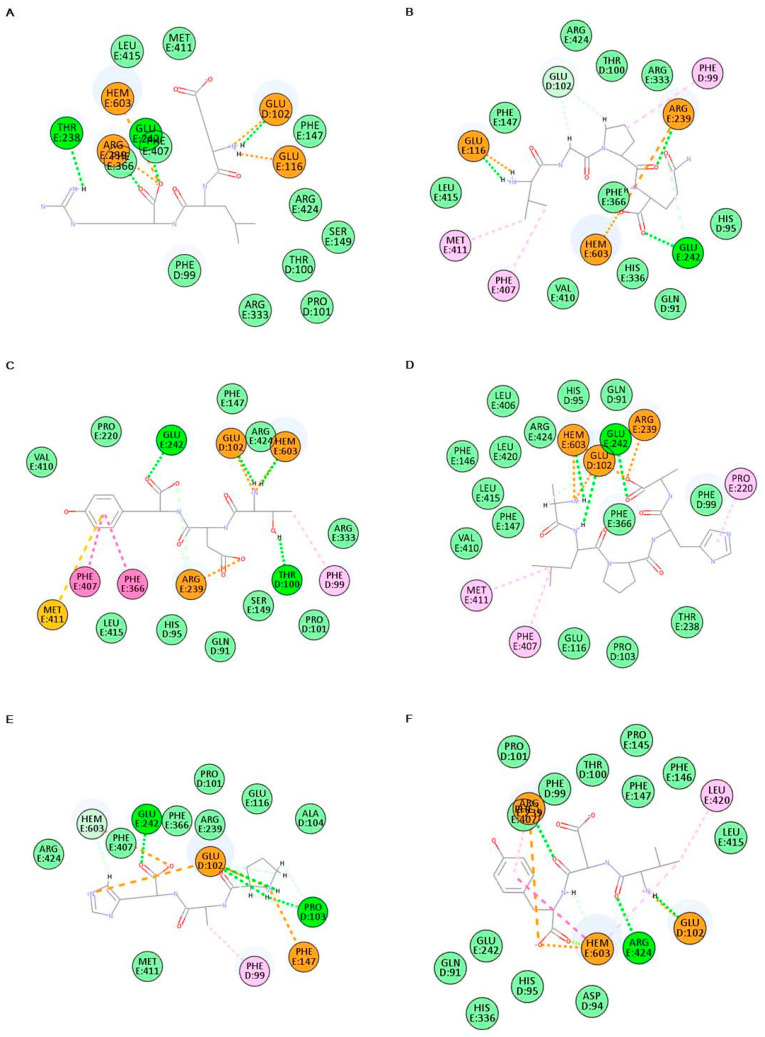

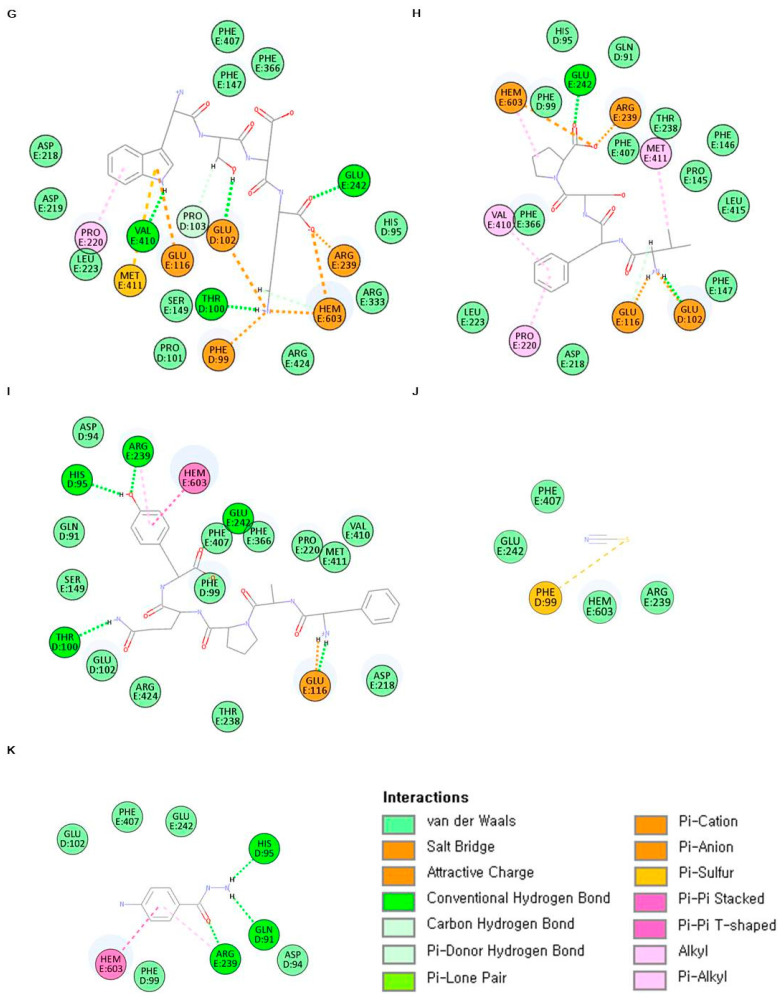
2D diagrams of bioactive peptides-MPO complexes. ELR (**A**), VGPQ (**B**), TDY (**C**), ALPAH (**D**), PAH (**E**), VDY (**F**), WSDK (**G**), VFSP (**H**), FAPQY (**I**), thiocyanate ion (**J**), and 4-aminobenzoic acid hydrazide (4-ABH) (**K**).

To verify the antioxidant activity of bioactive peptides purified from TVP-GFC-3, the biological network dynamics of bioactive peptides and MPO were simulated in a computational space, and its binding energies were compared with thiocyanate ion, a pseudohalide anion, and 4-aminobenzoic acid hydrazide (4-ABH), an inhibitor of MPO. In silico analysis was performed using the crystalline structure of MPO (PDB ID 7LAL) and 4-ABH (PubChem CID 21450). Bioactive peptide structures were drawn using a CDOCKER tool. Each amino acid of bioactive peptides forms diverse hydrogen and pi bonds; also, all bioactive peptides dock to the active site of MPO with a more stable binding energy than that of thiocyanate ion (Figure 5, Table 4). All of the bioactive peptides form the hydrogen bonds and/or pi bonds with a heme group. The activation of MPO is required a heme group as a cofactor [30].

**Figure 6:**
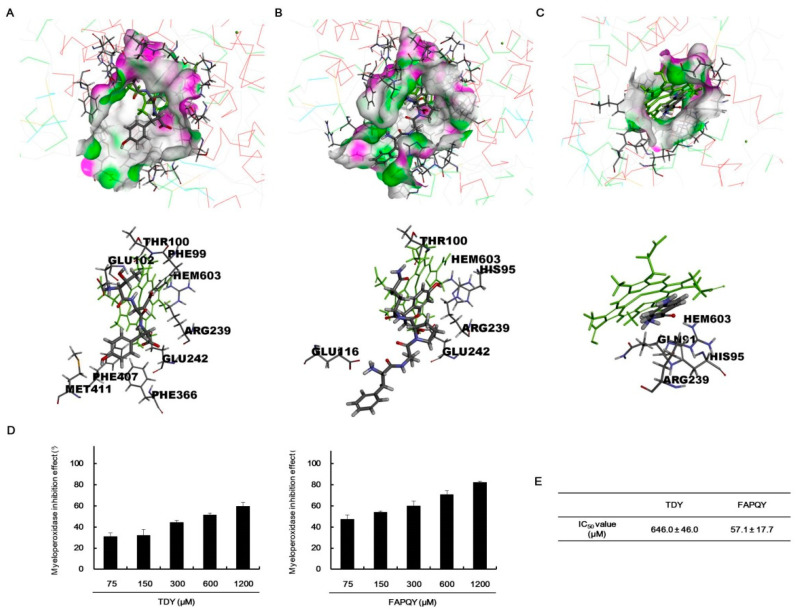
Myeloperoxidase (MPO) inhibition effects of TDY and FAPQY. (**A**–**C**) TDY, FAPQY, and 4-aminobenzoic acid hydrazide (4-ABH) are shown as a gray and red stick model. MPO is shown as a line model, and the active site of MPO is shown in the thin stick model. (**A**) The favorable hydrogen bond interactions of the TDY–MPO complex. (**B**) The favorable hydrogen bond interactions of the FAPQY–MPO complex. (**C**) The favorable hydrogen bond interactions of 4-ABH-MPO complex. (**D**) MPO inhibition effects of TDY and FAPQY in in vitro analysis. (**E**) IC_50_ values on MPO inhibition effect of TDY and FAPQY.

4-ABH is one of hydrazide with the formula H_2_NC_6_H_4_C(O)NHNH_2_ containing two amino groups and benzene ring. 4-ABH is docking to the active site of MPO with hydrogen bond and pi bond between the amino groups and benzene ring. Among the bioactive peptides, TDY and FAPQY having a benzene ring bound to MPO, with low binding energy of −368.111 and −387.049 kcal/mol, respectively. The top hit docking poses were presented as two-dimensional (2D) diagrams and three-dimensional (3D) to confirm the biological network dynamics of the complexes (Figure 5 and Figure 6). MPO was expressed as a line model, and the active site including a heme group (green part), was expressed as a thin stick model (Figure 6). TDY (Figure 6A) and FAPQY (Figure 6B) was shown as a gray and red stick model. The complexes displayed favorable hydrogen bond interactions, with the pink section as a donor and the green section as an acceptor (Figure 6A,B). The docking of TDY was performed through interaction with the active site, including a heme group and PHE99, THR100, GLU102, ARG239, GLU242, PHE366, PHE407, and MET411 (Figure 5C and Figure 6A). In addition, the docking of FAPQY was performed through interaction with the active site, including a heme group and HIS95, THR100, GLU116, ARG239, and GLU242 (Figure 5I and Figure 6B). Especially, FAPQY formed the similar binding pose with 4-ABH-MPO complex by combining as pi bonds between a benzene ring and a heme group and ARG239 (Figure 5K and Figure 6C). 

### 2.7. In Vitro Analysis of Antioxidant Peptides on Myeloperoxidase (MPO) Inhibition

To confirm the in silico prediction results on the docking of peptides to MPO, in vitro MPO inhibition effects of the peptides were assessed. Both TDY and FAPQY inhibited MPO in a concentration-dependent manner, and the IC_50_ values were calculated to be 646.0 ± 45.0 µM and 57.1 ± 17.7 µM, respectively (Figure 6D,E). These results indicated that these bioactive peptides possessed the values of the natural MPO inhibitors. MPO promotes oxidative stress by involving the generation of radicals [31]. As with many radical species, H_2_O_2_ can cause the oxidative stress, directly reacting the cells and/or indirectly inducing the production other radical species. Especially, the hypohalous acids were produced by the MPO with H_2_O_2_; these radicals cause the stronger oxidative stress. In addition, the scavenging of H_2_O_2_ affects to inhibit the MPO activities. Thus, the components having both H_2_O_2_ scavenging activity and MPO inhibition effects can be considered as valuable antioxidant. Therefore, the protamex-assisted extracts and peptide from *T. cornutus* having both H_2_O_2_ scavenging activity and MPO inhibition effects can be used as functional food components for human health.

## 3. Materials and Methods

### 3.1. Materials

*T. cornutus* was purchased from a fishing village in Taeheung in May 2019 (Jeju, Korea) and was washed thrice with tap water to remove salt, epiphytes, and sand attached to its surface. The viscera and muscle tissues were separated and carefully rinsed using fresh water and stored at −20 °C. Finally, the *T. cornutus* viscera and muscle tissues were freeze-dried and finely ground before hydrolysis. Commercial food-grade proteases, including alcalase 2.4 L FG, neutrase 0.8 L, flavourzyme 500 MG, and protamex, were purchased from Novo Co. (Novozyme Nordisk, Bagasvaerd, Denmark). Other proteases that contain pepsin, trypsin, α-chymotrypsin bromelain, and papain were purchased from Sigma Chemical Co. (St. Louis, MO, USA). The characterized peptide was synthesized by Anygen Co., LTD. (Gwangju, Korea) based on its amino acid sequence. Other chemicals and reagents used were of analytical grade.

### 3.2. Proximate Composition of T. cornutus

The proximate composition of *T. cornutus* was determined following the AOAC method [32]. Crude protein was determined using the Kjeldahl method, and crude lipid was performed using the Soxhlet method. In addition, moisture was determined by keeping the sample in a dry oven, and crude ash was prepared at 550 °C in a dry-type furnace. 

### 3.3. Amino Acid Profile

Amino acid compositions were analyzed according to a previously developed HPLC method [33]. The samples were added 30 mL of 6 N HCl and the mixtures were incubated for 24 h at 130 °C. The mixtures were filtered with 0.45 µm syringe filter, and used for HPLC sample. The HPLC system for analysis was consisted of an Ultimate3000 (Thermo Fisher Scientific, MA, USA) and FL detector 1260FLD (Agilent Technologies, Inc., Santa Clara, CA, USA). The analyses were carried out using a binary gradient mode. The mobile phase (A) was 40 mM sodium phosphate buffer (pH 7) and (B) was water:acetonitrile:methanol:water (10:45:45): 0 min, 5% B; 0–3 min, 5% B; 3–24 min, 55% B; 24–25 min, 80% B; 25–31 min, 80% B; 31–34.5 min, 5% B; 34.5–35 min, 5% B. The column temperature was kept at 40 °C, and the flow rate was 1.5 mL/min. An Inno C18 column (4.6 × 150 mm, 5 µm, YoungJin biochrom, Gyeonggi, Korea) was used. The chromatogram was detected using a fluorescence spectrophotometer at 340/450 nm (o-phthalaldehyde) and 266/305 nm (9-fluorenylmethyl chloroformate) and an absorbance at 338 nm.

### 3.4. Preparation of T. cornutus Enzyme-Assisted Extracts

*T. cornutus* viscera and muscle enzyme-assisted hydrolysis was performed according to the method used by Ko et al. [34] and Heo et al. [35]. Hydrolytic enzymes were prepared using four food-grade proteases (alcalase, flavourzyme, neutrase, and protamex), three digestive enzymes (pepsin, trypsin, and α-chymotrypsin), and two plant-derived digestive enzymes (bromelain and papain). Two grams of the dried ground *T. cornutus* viscera and muscle powder was homogenized with buffer (100 mL) and hydrolyzed with enzymes in a substrate/enzyme ratio of 100:1 (w/w). The pH of the homogenates was adjusted to its optimal pH value before enzymatic hydrolysis. Enzymatic reactions were performed for 24 h to achieve an optimal degree of enzymatic hydrolysis. Then, the extracts were boiled for 10 min at 100 °C in a water bath to inactivate the enzyme. Each extract was clarified by centrifugation (3500 rpm, 20 min at 4 °C) to remove the residue. All extracts were freeze-dried and kept at −20 °C. The yields of each *T. cornutus* viscera and muscle enzyme-assisted extracts were calculated as the percentage of dry weight compared with the hydrolyzed sample weight. Briefly, extract yields were determined by subtracting the dried weight of the residue from 1 mL of dried extracts and were expressed as a percentage.

### 3.5. Separation of Fractions and Radical Scavenging Properties

Radical scavenging properties were separated as previously described by Kang et al. [6]. The extracts showing antioxidant activities were dissolved in distilled water, loaded onto a Sephadex G-25 gel filtration column (2.5 × 75 cm), and equilibrated with distilled water. The column was eluted with distilled water at a flow rate of 1.0 mL/min. Elution peaks were detected at 280 nm.

### 3.6. Characterization of the Separated Antioxidant Properties

The molecular mass and amino acid sequence of the separated antioxidant properties from *T. cornutus* was determined using a MicroQ–TOF tandem mass spectrometer (Bruker Daltonics, 255748 Bremen, Germany) coupled with a nanoelectrospray ionization (ESI) source. The fraction dissolved in water was infused into the ESI source, and the molecular weight was determined by doubly charged (M + 2H)^2+^ state analysis in the mass spectrum. Following molecular weight determination, the peptides were automatically selected for fragmentation, and sequence information was obtained by tandem MS analysis.

### 3.7. H_2_O_2_ Scavenging Activity

H_2_O_2_ scavenging activity was determined according to the method of Müller [36]. One hundred microliters of 0.1-M phosphate buffer (pH 5.0) and twenty microliters of the sample solution were mixed in a 96-well plate. Twenty microliters of H_2_O_2_ was added to the mixture and then incubated at 37 °C for 5 min. After incubation, 30 μL of 1.25 mM ABTS and 30 μL of peroxidase (1 unit/mL) was added to the mixture and then incubated at 37 °C for 10 min. The absorbance was read with a microplate reader at 405 nm.

### 3.8. Cell Line and Cell Culture

HepG2 cells were purchased from the Korean Cell Line Bank (Seoul, Korea). HepG2 cells were cultured in RPMI-1640 medium, supplemented with 10% fetal bovine serum, 1% streptomycin (100 μg/mL), and penicillin (100 unit/mL) and maintained at 37 °C in a 5% CO_2_ incubator.

### 3.9. Determination of Cell Viability and ROS Generation in H_2_O_2_-Treated HepG2 Cells

Potential antioxidant activities were evaluated under H_2_O_2_-induced oxidative conditions. Briefly, HepG2 cells were plated in 96-well plates at a concentration of 1 × 10^5^ cells/mL and incubated for 24 h. After 24 h of incubation, the samples were treated before activating them with H_2_O_2_ (1 mM) for 1 h. After 24 h of incubation, cell viability was measured using the MTT assay [37]. The intracellular ROS scavenging activity was analyzed using the DCF-DA assay [38]. The HepG2 cells were seeded as shown before, treated with H_2_O_2_ and different concentrations of samples, and incubated for 24 h. After 24 h of incubation, 500 µg/mL of DCF-DA was added to each well. Finally, DCF-DA fluorescence was measured using a Synergy HT Multi-Detection Microplate Reader (BioTek Instruments, Winooski, VT, USA) at excitation and emission wavelengths of 485 and 535 nm, respectively.

### 3.10. In Silico Analysis of MPO Inhibition

For molecular docking studies, the crystal structures of MPO (ID: 7LAL) were provided by the Protein Data Bank. The structures of nine bioactive peptides derived from TVP were drawn using a CDOCKER tool. The docking of bioactive peptides to MPO was performed using the CDOCKER tool in Discovery Studio 2020 (Dassault Systemes, Velizy-Villacoublay, France). The simulation was performed as follows: (1) a 2D structure was converted into a 3D structure; (2) receptors were prepared, and the binding site was defined; and (3) the docking of compounds was performed using a CDOCKER tool [28]. The binding energies of the produced complexes were calculated to compare the optimal agents among the bioactive peptides, inhibitors, and existing ligand (thiocyanate ion). The docking poses of bioactive peptides to MPO were expressed as 2D diagrams and 3D crystalline structures.

### 3.11. MPO Inhibition Effect

MPO inhibition effects of the peptides were measured by using an MPO inhibitor screening assay kit (Abcam PLC, Cambridge, UK) following the instruction in the enclosed user manuals. Briefly, 10 µL of each peptide, 10 µL of 1.25 µL/mL MPO and 40 µl of assay buffer were mixed in a 96-well black plate. 50 µL of the peroxidation initiator solution was quickly added to all of the wells and then incubated for 5 min at RT. After incubation, the fluorescence intensity of the each well was read using an excitation wavelength of 530 nm and an emission wavelength of 590 nm.

### 3.12. Statistical Analysis

All data were represented as the mean ± standard deviation of three determinations. The statistical comparison of the mean values was performed by one-way ANOVA, followed by Tukey’s multiple comparisons test. Statistical significance was considered at *p* < 0.01.

## 4. Conclusions

By the sea food industrial activities, a considerable amount of fishery resources are discarded as processing leftovers including viscera. Thus, the possibility to recover such a material and convert it in a value-added product would be highly desirable. In Korea, *T. cornutus* muscle tissues are used in local foods, but most of the viscera tissues were discarded. *T. cornutus* viscera is a rich protein source, with more than 50% of protein contents composed of essential amino acids, such as histidine, threonine, valine, methionine, phenylalanine, isoleucine, leucine, and lysine. In addition, the potential antioxidant properties from *T. cornutus* viscera extracts possessed H_2_O_2_ scavenging activity and protective effects on oxidative stress in H_2_O_2_-treated HepG2 cells. The potential antioxidant properties were composed of nine bioactive peptides. In addition, in silico analysis predicted that the nine bioactive peptides inhibit peroxidase by interacting with the surface of MPO close to the active site. Especially, TDY and FAPQY showed the MPO inhibition effects with IC_50_ values of 646.0 ± 45.0 µM and 57.1 ± 17.7 µM, respectively. These results indicated that the potential antioxidant properties from *T. cornutus* viscera could be used for functional food components for human health.

## Figures and Tables

**Table 1:** The proximate composition of *T. cornutus* (% on dry weight).

	Viscera	Muscle
Proteins	52.68 ± 0.28	78.28 ± 2.23
Lipids	28.40 ± 1.20	10.90 ± 0.81
Moisture	1.03 ± 0.35	4.25 ± 3.66
Ash	14.79 ± 0.80	4.86 ± 0.40
Carbohydrates	3.12 ± 1.93	1.73 ± 1.85
Total	100	100

**Table 2:** The amino acids composition of *T. cornutus* (% of total amino acids).

	Viscera	Muscle
Aspartic acid	10.3 ± 0.0	9.5 ± 0.2
Glutamic acid	13.1 ± 0.2	16.4 ± 0.1
Serine	4.9 ± 0.1	4.7 ± 0.1
Histidine	1.8 ± 0.0	1.1 ± 0.1
Glycine	5.5 ± 0.4	8.8 ± 0.2
Threonine	5.2 ± 0.1	4.4 ± 0.0
Arginine	7.1 ± 0.5	9.6 ± 0.0
Alanine	5.0 ± 0.1	5.9 ± 0.0
Taurine	11.3 ± 0.1	8.1 ± 0.0
Tyrosine	3.5 ± 0.0	2.6 ± 0.0
Valine	4.4 ± 0.3	3.3 ± 0.2
Methionine	2.3 ± 0.1	2.2 ± 0.2
Phenylalanine	4.4 ± 0.1	2.8 ± 0.1
Isoleucine	3.8 ± 0.3	3.1 ± 0.2
Leucine	6.5 ± 0.1	6.3 ± 0.1
Lysine	4.8 ± 0.1	4.5 ± 0.9
Proline	6.2 ± 0.3	6.6 ± 1.9
Total	100	100

**Table 3:** Nine bioactive peptides from TVP-GFC-3.

Sample	Charge	m/z	Sequencing
TVP-GFC-3	1	417.25	ELR
	1	400.22	VGPQ
	1	398.16	TDY
	1	508.29	ALPAH
	1	324.17	PAH
	1	396.18	VDY
	1	535.25	WSDK
	1	449.24	VFSP
	1	625.30	FAPQY

**Table 4:** Docking results bioactive peptide-MPO complexes.

Sample	Characteristic of Peptide-MPO Complexes	
	Binding Energy (kcal/mol)	Main Bonding
ELR	−426.358	HEM603, GLU102, GLU116, THR238, ARG239, GLU242, PHE366, PHE407
VGPQ	−509.950	HEM603, PHE99, GLU102, GLU116, ARG239, GLU242, PHE366, PHE407, MET411
TDY	−368.111	HEM603, PHE99, THR100, GLU102, ARG239, GLU242, PHE366, PHE407, MET411
ALPAH	−360.686	HEM603, GLU102, PRO220, ARG239, GLU242, PHE407, MET411
PAH	−430.944	HEM603, PHE99, GLU102, PRO103, PHE147, GLU242
VDY	−398.554	HEM603, GLU102, ARG239, PHE407, LEU420, ARG424
WSDK	−340.875	HEM603, PHE99, THR100, GLU102, PRO103, GLU116, PRO220, ARG239, GLU242, VAL410, MET411
VFSP	−442.737	HEM603, GLU102, GLU116, PRO220, ARG239, GLU242, VAL410, MET411
FAPQY	−387.049	HEM603, HIS95, THR100, GLU116, ARG239, GLU242
Thiocyanate ion	−33.0451	PHE99
4-aminobenzoic acid hydrazide (4-ABH)	−74.8248	HEM603, GLN91, HIS95, ARG239

## Supplementary Materials

The following are available online at https://www.mdpi.com/article/10.3390/md19100567/s1. Figure S1: LC-MS/MS chromatogram of TVP-GFC-3. Supplementary Figures S2–S10: MS/MS sequencing of bioactive peptides.

## References

[1] Chung S.C., Lee J.J., Lee C.K. (1983). A study on the growth of Jeju Island’s Turban Shell, *Turbo cornutus*. Bull. Mar. Resour. Res. Inst..

[2] Chang D.S. (2002). Studies on the stock assessment and management of the Turban Shell, *Batillus cornutus* in Jeju Coastal water, Korea. Ph.D. Thesis.

[3] Chung S.C. (1976). Studies on the biometry of the *Turbo cornutus* solander in the Cheju coastal waters. Bull. Mar. Biol. Stn..

[4] Yoo J.T., Oh B.S., Chang D.S. (2011). Preference of adult top shell (*Batillus cornutus*) on specific marine algae in the coastal waters of Jeju Island. Korean J. Malacol..

[5] Zou Z., Chang H., Li H., Wang S. (2017). Induction of reactive oxygen species: An emerging approach for cancer therapy. Apoptosis.

[6] Kang N., Ko S.C., Samarakoon K., Kim E.A., Kang M.C., Lee S.C., Kim J., Kim Y.T., Kim J.S., Kim H. (2013). Purification of antioxidative peptide from peptic hydrolysates of Mideodeok (*Styela clava*) flesh tissue. Food Sci. Biotechnol..

[7] Sroka Z., Cisowski W. (2003). Hydrogen peroxide scavenging, antioxidant and anti-radical activity of some phenolic acids. Food Chem. Toxicol..

[8] Ielciu I., Mouithys-Mickalad A., Franck T., Angenot L., Ledoux A., Păltinean R., Cieckiewicz E., Etienne D., Tits M., Crişan G. (2019). Flavonoid composition, cellular antioxidant activity and (myelo) peroxidase inhibition of a *Bryonia alba* L.(Cucurbitaceae) leaves extract. J. Pharm. Pharmacol..

[9] Ulfig A., Leichert L.I. (2021). The effects of neutrophil-generated hypochlorous acid and other hypohalous acids on host and pathogens. Cell. Mol. Life Sci..

[10] Gülçin Ý., Elias R., Gepdiremen A., Boyer L., Köksal E. (2007). A comparative study on the antioxidant activity of fringe tree (*Chionanthus virginicus* L.) extracts. Afr. J. Biotechnol..

[11] Rees M.D., Bottle S.E., Fairfull-Smith K.E., Malle E., Whitelock J.M., Davies M.J. (2009). Inhibition of myeloperoxidase-mediated hypochlorous acid production by nitroxides. Biochem. J..

[12] Pap R., Pandur E., Jánosa G., Sipos K., Agócs A., Deli J. (2021). Lutein exerts antioxidant and anti-Inflammatory effects and influences iron utilization of BV-2 microglia. Antioxidants.

[13] Ozogul F., Cagalj M., Šimat V., Ozogul Y., Tkaczewska J., Hassoun A., Kaddour A.A., Kuley E., Rathod N.B., Phadke G.G. (2021). Recent developments in valorisation of bioactive ingredients in discard/seafood processing by-products. Trends Food Sci. Technol..

[14] Pal G.K., Suresh P.V. (2016). Sustainable valorisation of seafood by-products: Recovery of collagen and development of collagen-based novel functional food ingredients. Innov. Food Sci. Emerg. Technol..

[15] Sila A., Bougatef A. (2016). Antioxidant peptides from marine by-products: Isolation, identification and application in food systems. A review. J. Funct. Foods.

[16] Xu D.P., Li Y., Meng X., Zhou T., Zhou Y., Zheng J., Zhang J.J., Li H.B. (2017). Natural antioxidants in foods and medicinal plants: Extraction, assessment and resources. Int. J. Mol. Sci..

[17] Rustad T., Storrø I., Slizyte R. (2011). Possibilities for the utilisation of marine by-products. Int. J. Food Sci. Technol..

[18] Arvanitoyannis I.S., Kassaveti A. (2008). Fish industry waste: Treatments, environmental impacts, current and potential uses. Int. J. Food Sci. Technol..

[19] Kim S.K., Venkatesan J. (2014). Introduction to Seafood Processing by-Products. Seafood Processing by-Products.

[20] Shahidi F., Varatharajan V., Peng H., Senadheera R. (2019). Utilization of marine by-products for the recovery of value-added products. J. Food Bioact..

[21] Klomklao S., Benjakul S. (2017). Utilization of tuna processing byproducts: Protein hydrolysate from skipjack tuna (*Katsuwonus pelamis*) viscera. J. Food Process. Preserv..

[22] Oh J.Y., Kim E.A., Lee H., Kim H.S., Lee J.S., Jeon Y.J. (2019). Antihypertensive effect of surimi prepared from olive flounder (*Paralichthys olivaceus*) by angiotensin-I converting enzyme (ACE) inhibitory activity and characterization of ACE inhibitory peptides. Process. Biochem..

[23] Regenstein J., Zhou P. (2007). Collagen and gelatin from marine by-products. Maximising the Value of Marine by-Products.

[24] Kim S.K., Mendis E. (2006). Bioactive compounds from marine processing byproducts—A review. Food Res. Int..

[25] Jang M.S., Jang J.R., Park H.Y., Yoon H.D. (2010). Overall composition, and levels of fatty acids, amino acids, and nucleotide-type compounds in wild abalone *Haliotis gigantea* and cultured abalone *Haliotis discus* hannai. Korean J. Food Preserv..

[26] Xia T., Yao J., Zhang J., Zheng Y., Song J., Wang M. (2017). Protective effects of Shanxi aged vinegar against hydrogen peroxide-induced oxidative damage in LO2 cells through Nrf2-mediated antioxidant responses. RSC Adv..

[27] Kang N., Lee J.H., Lee W., Ko J.Y., Kim E.A., Kim J.S., Heu M.S., Kim G.H., Jeon Y.J. (2015). Gallic acid isolated from *Spirogyra* sp. improves cardiovascular disease through a vasorelaxant and antihypertensive effect. Environ. Toxicol. Pharmacol..

[28] Kang N., Ko S.C., Kim H.S., Yang H.W., Ahn G., Lee S.C., Lee T.G., Lee J.S., Jeon Y.J. (2020). Structural evidence for antihypertensive effect of an antioxidant peptide purified from the edible marine animal *Styela clava*. J. Med. Food.

[29] Wu G., Robertson D.H., Brooks C.L., Vieth M. (2003). Detailed analysis of grid-based molecular docking: A case study of CDOCKER—A CHARMm-based MD docking algorithm. J. Comput. Chem..

[30] Heinecke J.W., Li W., Francis G.A., Goldstein J.A. (1993). Tyrosyl radical generated by myeloperoxidase catalyzes the oxidative cross-linking of proteins. J. Clin. Investig..

[31] Baali N., Mezrag A., Bouheroum M., Benayache F., Benayache S., Souad A. (2020). Anti-inflammatory and Antioxidant Effects of *Lotus corniculatus* on Paracetamol-induced Hepatitis in Rats. Anti-Inflamm. Anti-Allergy Agents Med. Chem..

[32] Helrich K. (1990). Official Methods of Analysis of the Association of Official Analytical Chemists.

[33] Herbert P., Santos L., Alves A. (2001). Simultaneous quantification of primary, secondary amino acids, and biogenic amines in musts and wines using OPA/3-MPA/FMOC-CI fluorescent derivatives. J. Food Sci..

[34] Ko S.C., Lee J.K., Byun H.G., Lee S.C., Jeon Y.J. (2012). Purification and characterization of angiotensin I-converting enzyme inhibitory peptide from enzymatic hydrolysates of *Styela clava* flesh tissue. Process Biochem..

[35] Heo S.J., Park E.J., Lee K.W., Jeon Y.J. (2005). Antioxidant activities of enzymatic extracts from brown seaweeds. Bioresour. Technol..

[36] Müller H.E. (1985). Detection of hydrogen peroxide produced by microorganisms on an ABTS peroxidase medium. Zent. Bakteriol..

[37] Han E.J., Fernando I.P.S., Kim E.A., Kim J., Jung K., Kim S.Y., Cha S.H., Kim K.N., Heo S.J., Ahn G. (2020). 5-Bromo-3, 4-dihydroxybenzaldehyde from *Polysiphonia morrowii* attenuate IgE/BSA-stimulated mast cell activation and passive cutaneous anaphylaxis in mice. Biochem. Pharmacol..

[38] Ding Y., Jiratchayamaethasakul C., Kim E.A., Kim J., Heo S.J., Lee S.H. (2018). Hyaluronidase inhibitory and antioxidant activities of enzymatic hydrolysate from Jeju Island red sea cucumber (*Stichopus japonicus*) for novel anti-aging cosmeceuticals. J. Mar. Biosci. Biotechnol..

