# Supplementary material for: Identifying Potential Antioxidant Properties from the Viscera of Sea Snails (Turbo cornutus)
Source: Mar Drugs. 2021 Oct 13;19(10):567. doi: 10.3390/md19100567 (PMC8539058; doi:10.3390/md19100567)
Supplement: Supplementary file 1 [file marinedrugs-19-00567-s001.zip › marinedrugs-1341701-supplementary.pdf]

# Supplementary materials

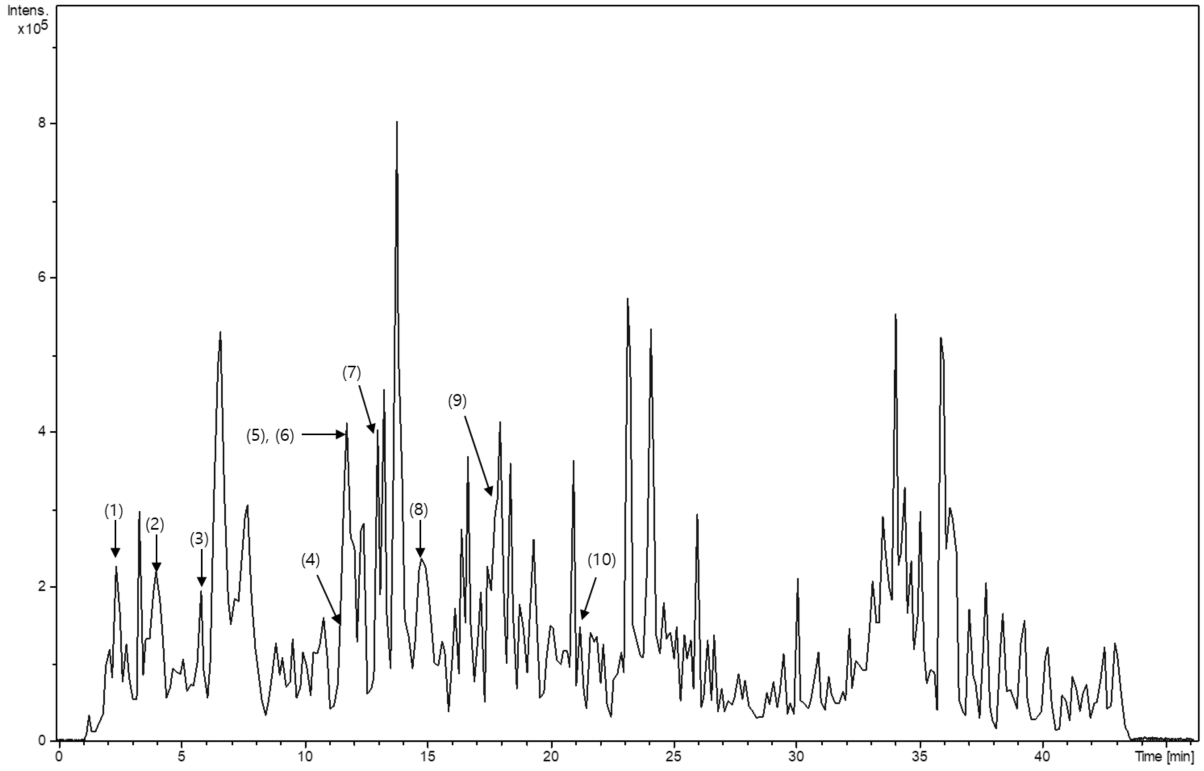


**Figure. S1.** LC-MS/MS chromatogram of TVP-GFC-3

417.25[M+H] : ELR

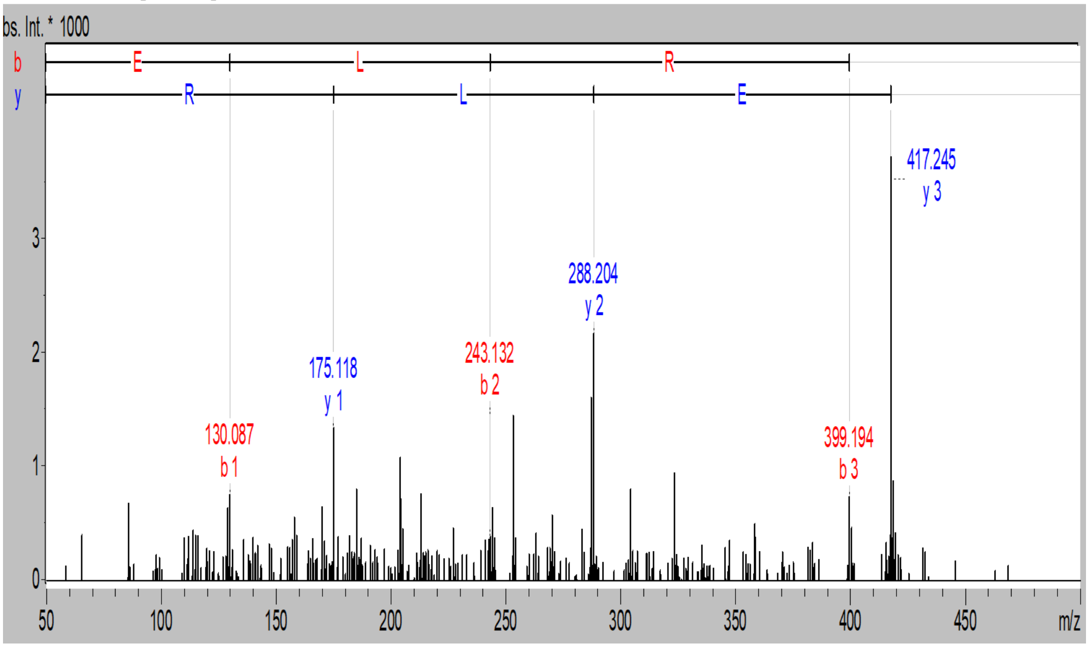


**Figure. S2.** MS/MS sequencing of bioactive peptide, ELR

400.22[M+H] : VGPQ

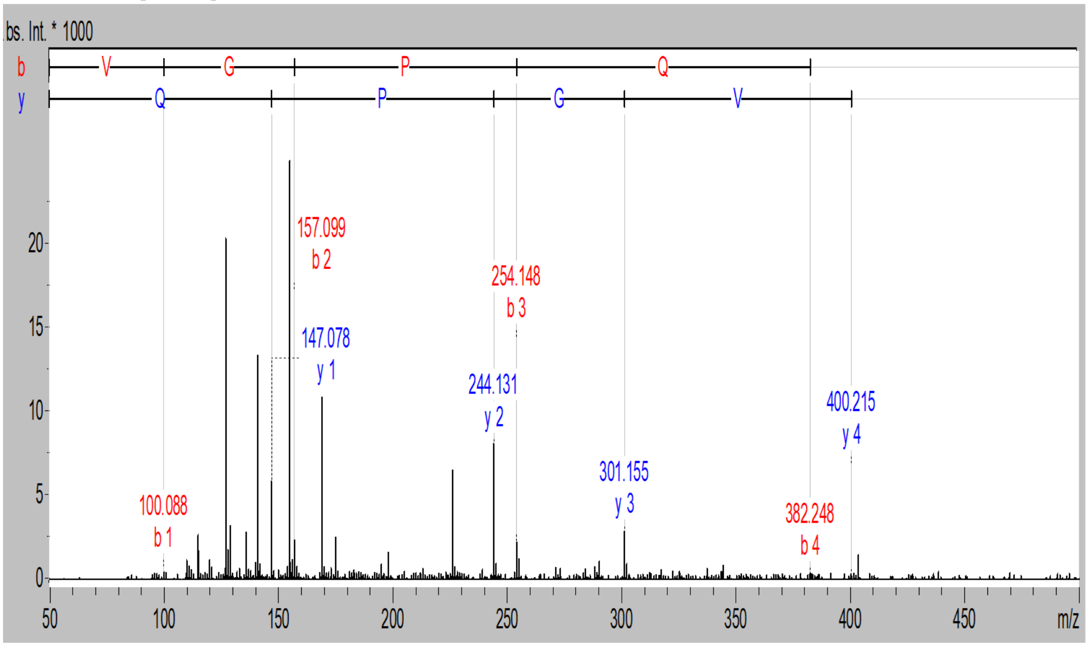


**Figure. S3.** MS/MS sequencing of bioactive peptide, VGPQ

398.16[M+H] : TDY

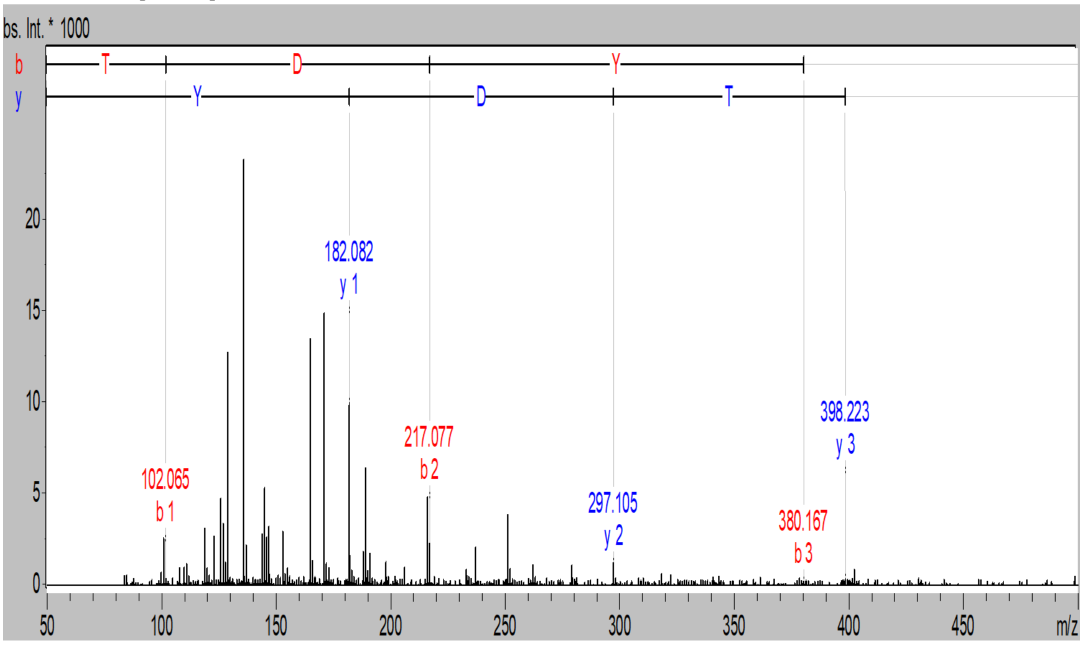


**Figure. S4.** MS/MS sequencing of bioactive peptide, TDY

508.29[M+H] : ALPAH

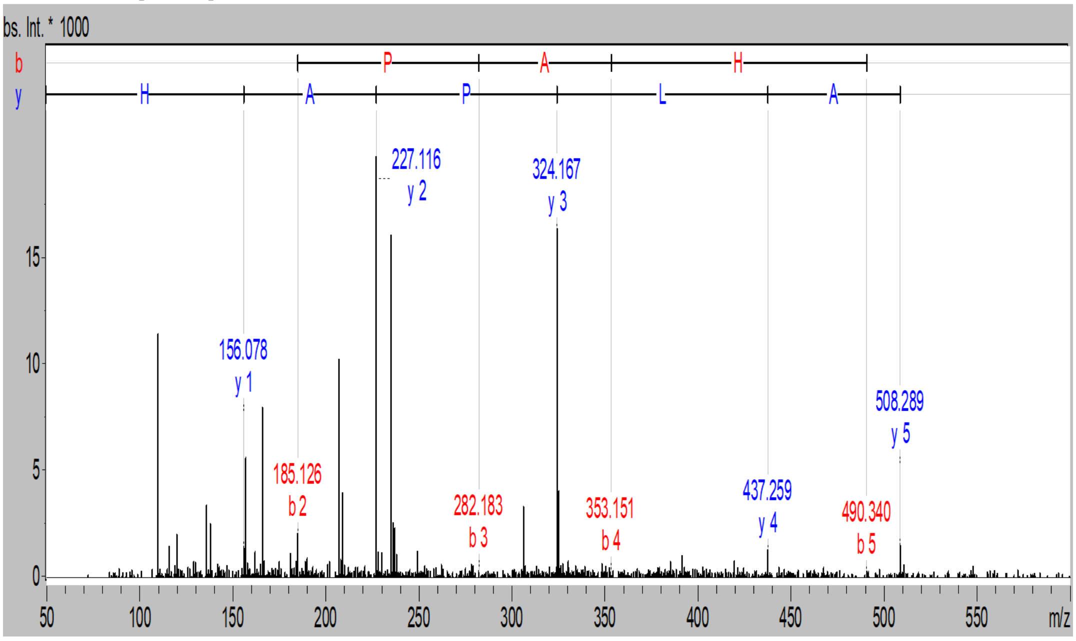


**Figure. S5.** MS/MS sequencing of bioactive peptide, ALPAH

324.17[M+H] : PAH

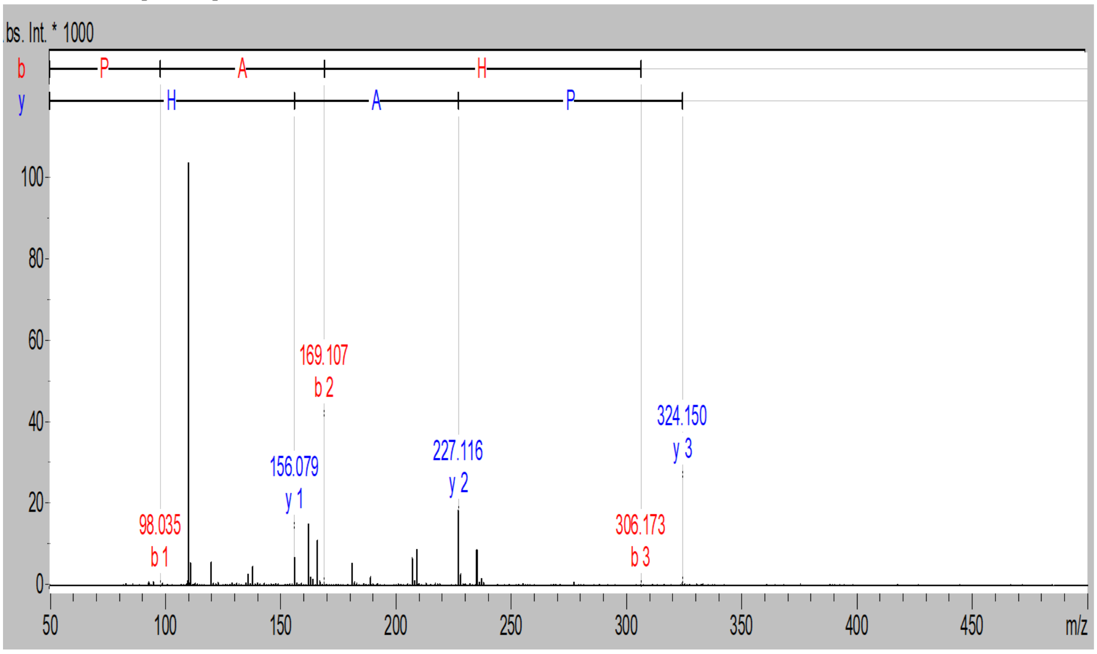


**Figure. S6.** MS/MS sequencing of bioactive peptide, PAH

396.18[M+H] : VDY

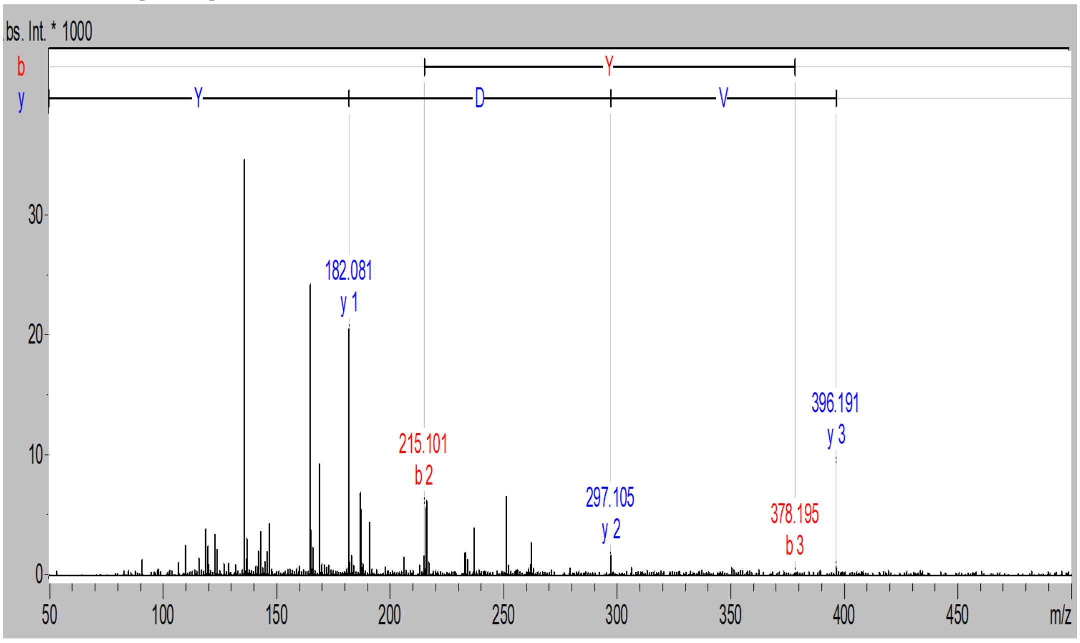


**Figure. S7.** MS/MS sequencing of bioactive peptide, VDY

535.25[M+H] : WSDK

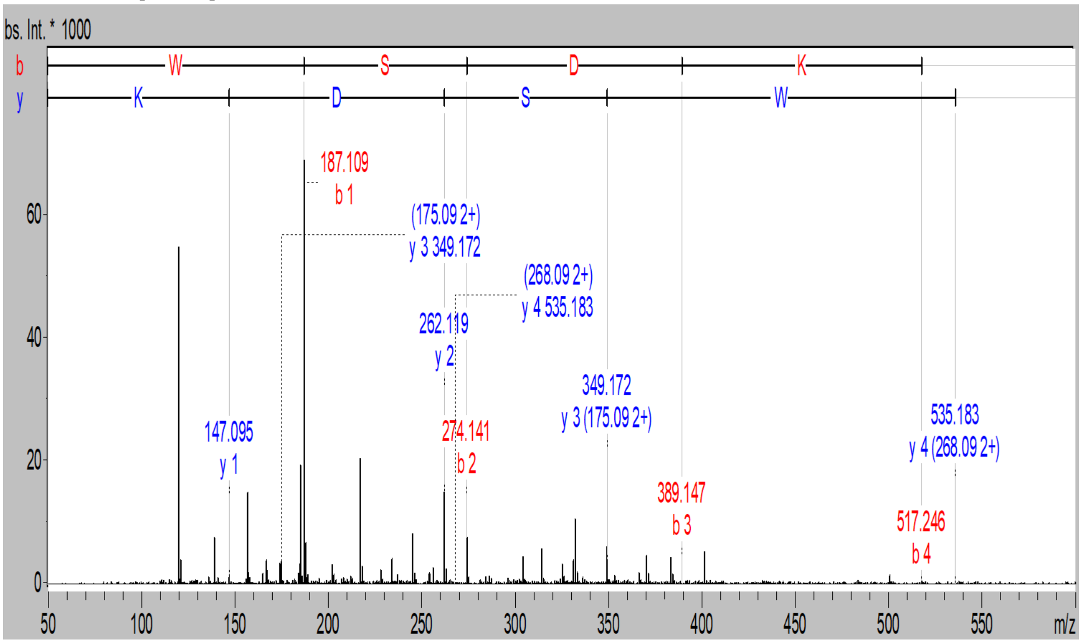


**Figure. S8.** MS/MS sequencing of bioactive peptide, WSDK

449.24[M+H] : VFSP

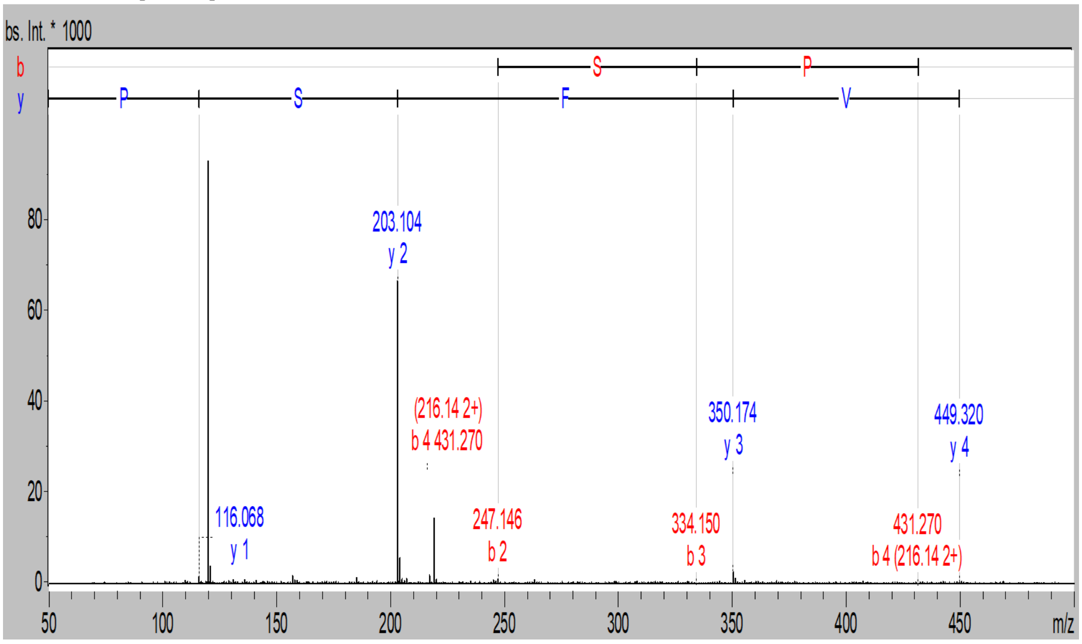


**Figure. S9.** MS/MS sequencing of bioactive peptide, VFSP

625.30[M+H] : FAPQY

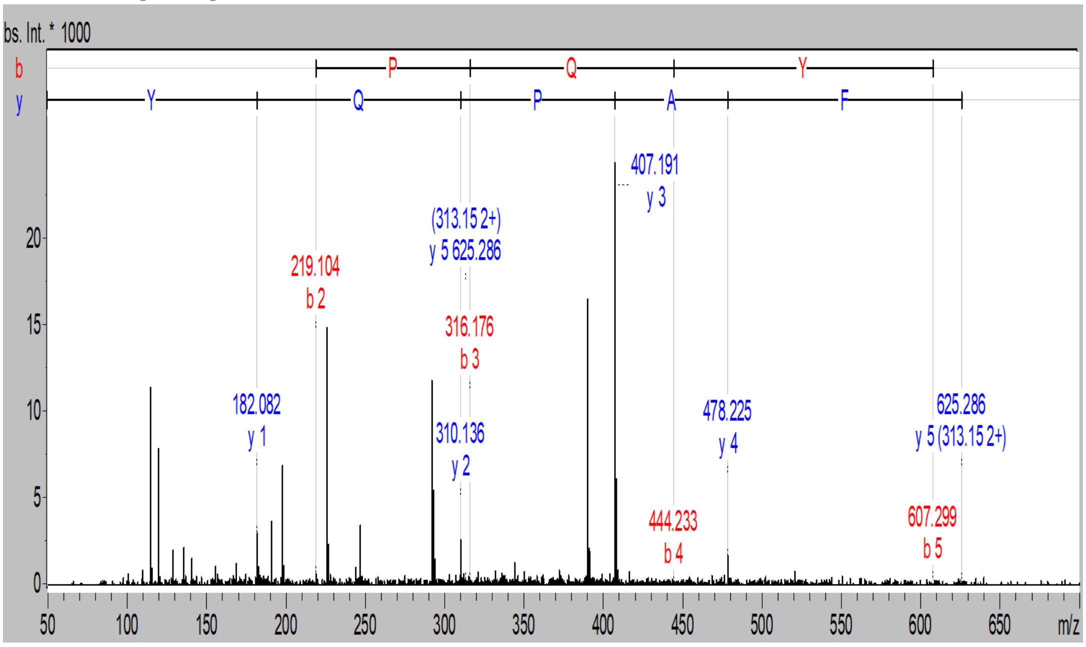


**Figure. S10.** MS/MS sequencing of bioactive peptide, FAPQY
